# Large-scale genomic analysis of global *Klebsiella pneumoniae* plasmids reveals multiple simultaneous clusters of carbapenem-resistant hypervirulent strains

**DOI:** 10.1186/s13073-023-01153-y

**Published:** 2023-01-19

**Authors:** Anton Spadar, João Perdigão, Susana Campino, Taane G. Clark

**Affiliations:** 1 Faculty of Infectious and Tropical Diseases, London School of Hygiene & Tropical Medicine, London, UK; 2 Research Institute for Medicines (iMed.ULisboa), Faculdade de Farmácia, Universidade de Lisboa, Lisboa, Portugal; 3 Faculty of Epidemiology and Population Health, London School of Hygiene and Tropical Medicine, London, UK

**Keywords:** AMR, Carbapenem, Hypervirulence, *Klebsiella*

## Abstract

**Background:**

*Klebsiella pneumoniae* (Kp) Gram-negative bacteria cause nosocomial infections and rapidly acquire antimicrobial resistance (AMR), which makes it a global threat to human health. It also has a comparatively rare hypervirulent phenotype that can lead to severe disease in otherwise healthy individuals. Unlike classic Kp, canonical hypervirulent strains usually have limited AMR. However, after initial case reports in 2015, carbapenem-resistant hypervirulent Kp has increased in prevalence, including in China, but there is limited understanding of its burden  in other geographical regions.

**Methods:**

Here, we examined the largest collection of publicly available sequenced Kp isolates (*n*=13,178), containing 1603 different sequence types (e.g. ST11 15.0%, ST258 9.5%), and 2174 (16.5%) hypervirulent strains. We analysed the plasmid replicons and carbapenemase and siderophore encoding genes to understand the movement of hypervirulence and AMR genes located on plasmids, and their convergence in carbapenem-resistant hypervirulent Kp.

**Results:**

We identified and analysed 3034 unique plasmid replicons to inform the epidemiology and transmission dynamics of carbapenem-resistant hypervirulent Kp (*n*=1028, 7.8%). We found several outbreaks globally, including one involving ST11 strains in China and another of ST231 in Asia centred on India, Thailand, and Pakistan. There was evidence of global flow of Kp, including across multiple continents. In most cases, clusters of Kp isolates are the result of hypervirulence genes entering classic strains, instead of carbapenem resistance genes entering canonical hypervirulent ones.

**Conclusions:**

Our analysis demonstrates the importance of plasmid analysis in the monitoring of carbapenem-resistant and hypervirulent strains of Kp. With the growing adoption of omics-based technologies for clinical and surveillance applications, including in geographical regions with gaps in data and knowledge (e.g. sub-Saharan Africa), the identification of the spread of AMR will inform infection control globally.

**Supplementary Information:**

The online version contains supplementary material available at 10.1186/s13073-023-01153-y.

## Background

*Klebsiella pneumoniae* (Kp) is a Gram-negative pathogen increasingly capable of causing severe organ and life-threatening disease. Kp is classified into two main virulence phenotypes: hypervirulent (hvKp) and classical (non-hvKp). Classical Kp is the most common phenotype and normally a nosocomial infection that occurs in patients with additional co-morbidities [1]. Less common is hvKp, which is characterised by invasive infection within the community setting in healthy individuals and a rapid metastatic spread. Epidemiologically, hvKp is more common in East and Southeast Asia, but it is also an emerging threat in Europe. HvKp associated with carbapenemase-producing clones is particularly concerning [1–4] as it will make infection control more difficult. It is therefore important to use genomic data and analyses from local, regional, and global studies to monitor its emergence and spread.

The main driver of carbapenem resistance in Kp is the acquisition of genes that encode carbapenemases such as KPC, NDM, some variants of OXA and others [5, 6]. The encoding genes are usually located on small mobile genetic elements such as transposons or insertion sequences. These elements are themselves usually embedded in plasmids, which mediate the transfer of genes between bacterial cells via horizontal gene transfer [7–9].

Similarly, hypervirulence is normally associated with aerobactin (*iuc*) and salmochelin (*iro*) gene loci carried on plasmids. The *iro* and *iuc* loci are frequently accompanied by additional genes (e.g. *rmpA*, *rmpA2*, and *rmpC*) associated with a hypermucoviscous capsule phenotype [10, 11]. K1 and K2 are the dominant capsular genotypes among hvKp [12]. The *iuc* and *iro* loci usually reside on large (>200kbp) plasmids, but are occasionally located on chromosomes via integrated chromosomal elements [1].

Since reports of carbapenem-resistant hypervirulent Kp (CRhvKp) in a tertiary hospital in Beijing in 2015 [13], the cases have steadily increased in number and geographic range [14–17]. The incidence of CRhvKp in China was estimated at ~13% based on the presence of *rmpA*, *rmpA2* or *iutA* biomarkers [14], but estimates vary regionally within the country. Another large-scale study in China reported that 36% of screened carbapenem-resistant (CRKp) strains carried the hypervirulence-associated siderophore aerobactin locus [17]. Other regional studies focused on Singapore [18], India [19] and the USA [20], while the global landscape has also been investigated [21]. Our work extends the previous reports by exploring in detail the plasmid replicons associated with the CRhvKp genotype, globally. With increased human mobility and volumes of trade and tourism, knowledge of the global genomic diversity of plasmids underlying CRhvKp strains will assist with monitoring the emergence and spread of CRhvKp infections and inform their clinical management.

In this study, we analysed plasmid replicons and carbapenemase and siderophore encoding genes across 12,468 geographically diverse Kp isolates to understand the movement of hypervirulence and antimicrobial resistance genes on plasmids, and their convergence in CRhvKp. Despite a reliance on using all available Kp assemblies instead of a targeted dataset, we found a growing prevalence of CRhvKp assigned to nine clusters, seven of which are statistically robust. These clusters included two previously reported outbreaks identified in China [14, 17, 21, 22]; however, they also include additional outbreaks with one spanning Asia, Africa, and Europe. Our findings demonstrate the utility of large-scale data for understanding the epidemiology of CRhvKp, with the potential to assist to clinical and surveillance investigations.

## Methods

All publicly available assemblies labelled as Kp (*n*=13,178) in the NCBI RefSeq database (as of September 2021) were downloaded [23]. Kleborate software (v2.2.0) was used to confirm species, identify sequence types (ST), find antimicrobial resistance (AMR) and virulence genes, and determine capsular and O-antigen types [21] (Additional file 1: Fig. S1, Additional file 2: Data S1). Isolates were classified with a hypervirulent Kp (hvKp) genotype if they contained either aerobactin (*iuc*), salmochelin (*iro*) or both gene loci [10, 11]. Carbapenem resistance can occur without carbapenemase encoding genes, but we conservatively defined a CRKp genotype as isolates that carry a carbapenemase encoding gene. Plasmid replicons were identified using PlasmidFinder software (v2.1.1) [24] with default cut-offs (≥60% coverage and ≥90% identity). The isolate data included complete, contig and scaffold level assemblies.

PlasmidFinder compares assemblies to a database of nucleotide sequences of genes encoding replication control and initiation proteins. The software returns assembly sub-sequences (not necessarily whole contigs) that pass similarity thresholds as either nucleotide sequences or their hashes. The returned sub-sequences were summarised in a binary data matrix (1 = present, 0 absent), where the rows represented individual isolates, and columns (*n*=3034) were unique replicon nucleotide sequences represented by their hashes. The hashes are more convenient for this task due to their shorter length (32 characters) compared to full nucleotide sequences, the longest of which was 1029nt. This binary matrix was used to calculate the *Russell-Rao* distance between pairs of isolates as implemented in sklearn software (v0.24.2) [25]. For a pair of samples *u* and *v*, the distance is defined as: $\frac{N-\sum_{i=1}^N\left(1\ if\ u\left[i\right]=v\left[i\right]=1\ else\ 0\right)\ }{N}$, where *N* is the total number of features. In the context of presence/absence data, the Russell-Rao distance has an advantage over the Jaccard metric, as it takes the total number of features (*N*) into account. Further, it is an improvement over the Euclidean metric because it returns a distance of one to those samples that have none of the features, instead of zero under the Euclidean metric.

We used the UMAP (v0.5.1) algorithm to project the full 3034-dimensional binary matrix into two-dimensional space [25–27]. UMAP aims to project multi-dimensional data into fewer dimensions while preserving some global and local data topology. The approach has similarity to principal components analysis (PCA) and multi-dimensional scaling (MDS or PCoA), where the former uses a covariance matrix and the latter uses a matrix of pairwise distances. Neither PCA nor MDS are intended for binary matrices, though MDS can accommodate ordinal data [28, 29]. UMAP belongs to the family of manifold learning algorithms, which perform well at dimensional reduction of binary data [30–32]. We applied HDBSCAN software (v0.81) (min_cluster_size=10 and cluster_selection_epsilon=0.5) to the 2-D projection generated by UMAP to identify clusters of isolates [33]. HDSCAN is a density-based clustering algorithm which does not require all data samples to be part of a cluster, and we have labelled such isolates as “unassigned”. In our context, the algorithm identifies how data from the UMAP 2-D projection is distributed in space, and determines gaps in density between groups of isolates. The minimum cluster size (“min_cluster_size”) parameter determines the density gap. The data points lying in regions of the same density are defined as clusters [33]. This adaptive approach to density, as opposed to a cut-off determined approach of DBSCAN, is the distinguishing feature of HDBSCAN. If, based on cluster density, a sample is too far to be part of the cluster, it becomes an outlier [33].

Unlike PCA and MDS, UMAP is a stochastic algorithm; so we assessed the robustness of clustering based on UMAP projection. For this purpose, we created a Monte Carlo simulation by repeating the UMAP projection and cluster detection 500 times. The clusters identified in each of 500 iterations formed columns of a data matrix, which was itself projected using UMAP with a Hamming distance metric. The clusters in this projection were again determined using HBSCAN (Additional file 1: Fig. S1). These latter clusters aggregate 500 individual runs, and to determine the consistency of aggregated versus each underlying cluster, we performed chi-squared tests, implemented in sklearn software. We labelled the aggregate cluster as robust if less than 1% of chi-squared test *p*-values were below 10^-20^ (Additional file 1: Fig. S1).

We also compared replicons found in our dataset to those characterised in 35 historic bacterial isolates [34] (Murray collection) sourced between the years 1920 and 1949 and classified as Kp based on Kleborate software typing.

## Results

### Isolates and genotypes

While all isolates (*n*=13,178) were Kp according to metadata, Kleborate screening identified them as Kp (*n*=11,820; 90%), *K. quasipneumoniae* (*n*=604, 5%), *K. variicola* (*n*=428; 3%), *K. aerogenes* (*n*=299; 2%) and other subspecies of *Klebsiella* (*n*=27; 0.2%), i.e. some isolates may have incorrect species data in the NCBI database (Additional file 1: Fig. S1). We did not restrict the analysis to *K. pneumoniae sensu stricto*, but we removed 710 isolates in which PlasmidFinder did not identify any replicons. These removed isolates covered 331 STs, with ST3910 (*n*=21; 3%) being most frequent. They also had a high abundance of non Kp *sensu stricto* (*K. quasipneumoniae n*=140, 19.7%; *K. aerogenes n*=115, 16.2%; *K. variicola* (*n*=109, 15.4%). Notably, these isolates had limited AMR carriage with only 9 isolates carrying carbapenemase encoding genes, among which *bla*_*OXA-48*_ was the most frequent (*n*=4). There were also few fluroquinolone *gyrA* mutations (*n*=65/710) and aminoglycoside resistance enzymes (*n*=35/710). Only four isolates carried an aerobactin locus. However, 20 isolates had a truncated *iro3* locus and 109 *K. aerogenes* samples had a chromosomally carried salmochelin locus.

The final isolate dataset (*n*=12,468) contained 1603 different STs (based on 7 chromosomal loci) with the most frequent being ST11 (15%), ST258 (10%), ST15 (4%), ST512 (4%) ST307 (3%) and ST147 (3%) (Additional file 1: Fig. S1). The number of hypervirulent strains was 1881 (15%). The two canonical hypervirulent strains ST23 and ST86 accounted for 1.8% and 0.7%, respectively. The study had global representation, with isolates from 103 countries, including China (20%), USA (14%), Italy (7%), UK (6%), Thailand (4%), and Germany (4%); however, only 2% of all isolates were from sub-Saharan Africa. While the convenience nature of the sampling may make the dataset unsuitable for the estimation of the prevalence of CRhvKp genotypes, the large sample size presents a likely accurate assessment of regional and temporal trends.

### Replicons

**Fig. 1:**
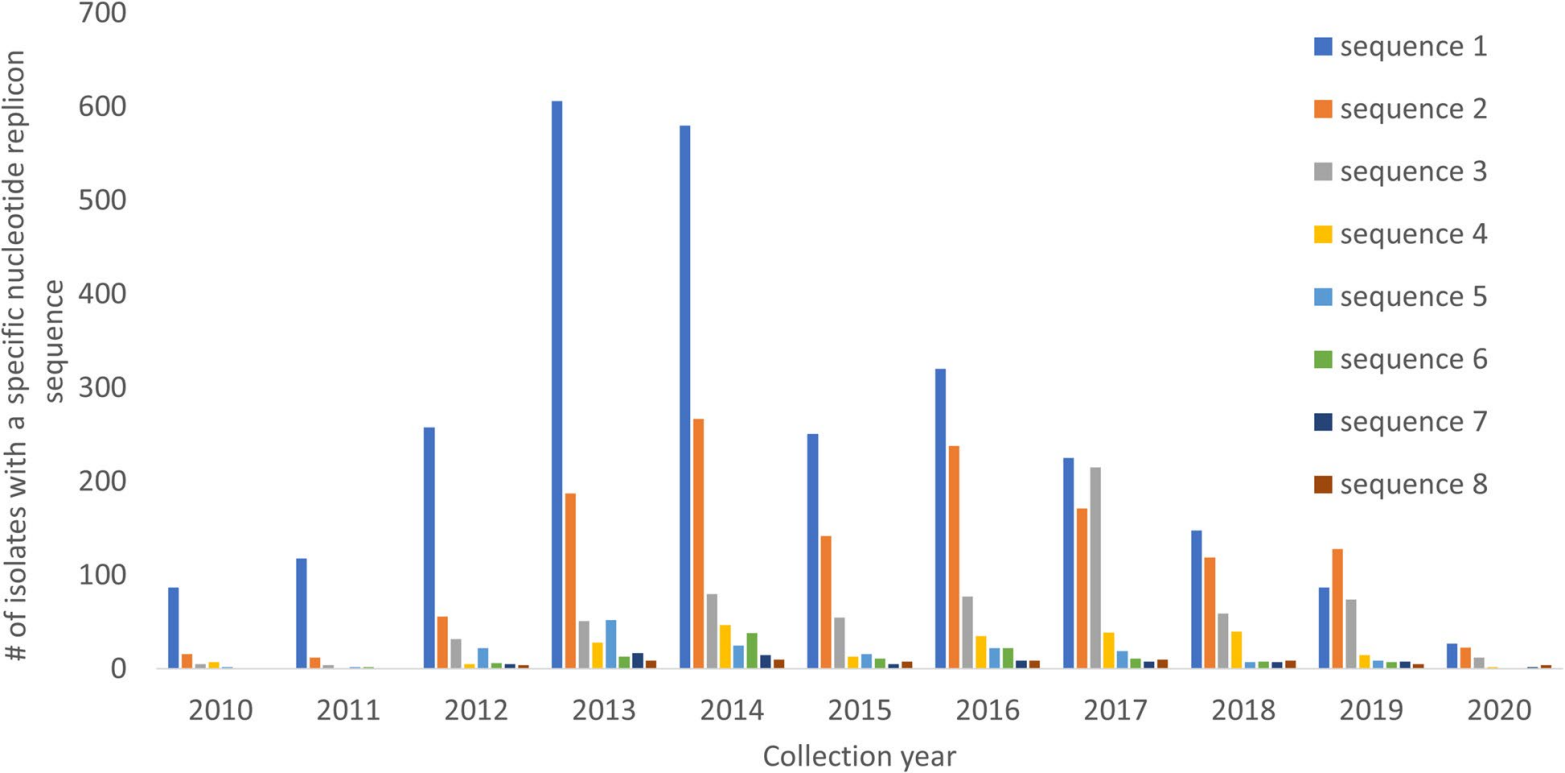
Frequency of the main nucleotide sequences from the IncFIB(K) replicon family. The eight nucleotide sequences are provided in Additional file 2: Data S2

An important cornerstone of our analysis is the distinction between the replicon family or name (e.g. IncFII(K), IncFIB(K) and ColRNAI) and the underlying unique nucleotide sequences of the replicons belonging to a specific family. For example, across the 12,468 isolates, there were 3034 unique replicon nucleotide sequences, of which 1096 occurred in more than one isolate. The most frequent replicon nucleotide sequence was the PlasmidFinder reference version of IncFIB(K), which occurred in 3123 (24%) isolates. However, the replicons from an IncFIB(K) family occurred 7052 times with eight distinct sequences occurring in more than 100 Kp isolates (Fig. 1). The family and nucleotide sequence diversity are summarised in Additional file 2: Data S2.

The average number of replicons per isolate was 4.6 (range: 1 to 29). A total of 6783 isolates (55%) with identified replicons had carbapenemase encoding genes, and 1881 (15%) had either *iuc* or *iro* loci. A total of 1028 (8%) isolates with identified replicons had both carbapenemase encoding and hypervirulence (CRhvKp) loci (Additional file 1: Fig. S1). Therefore, the numbers of isolates determined to be CRhvKp, CRKp (non- hvKp) and hvKp (non-CRKp) were 1028 (8%), 5755 (46%), and 853 (7%), respectively (Additional file 1: Fig. S1). The majority of the CRhvKp were from China (*n*=696) representing 27% of isolates from that country with isolates collected in Russia (*n*=64), Thailand (*n*=44), Italy (*n*=39) and India (*n*=25) being the next most common.

**Fig. 2:**
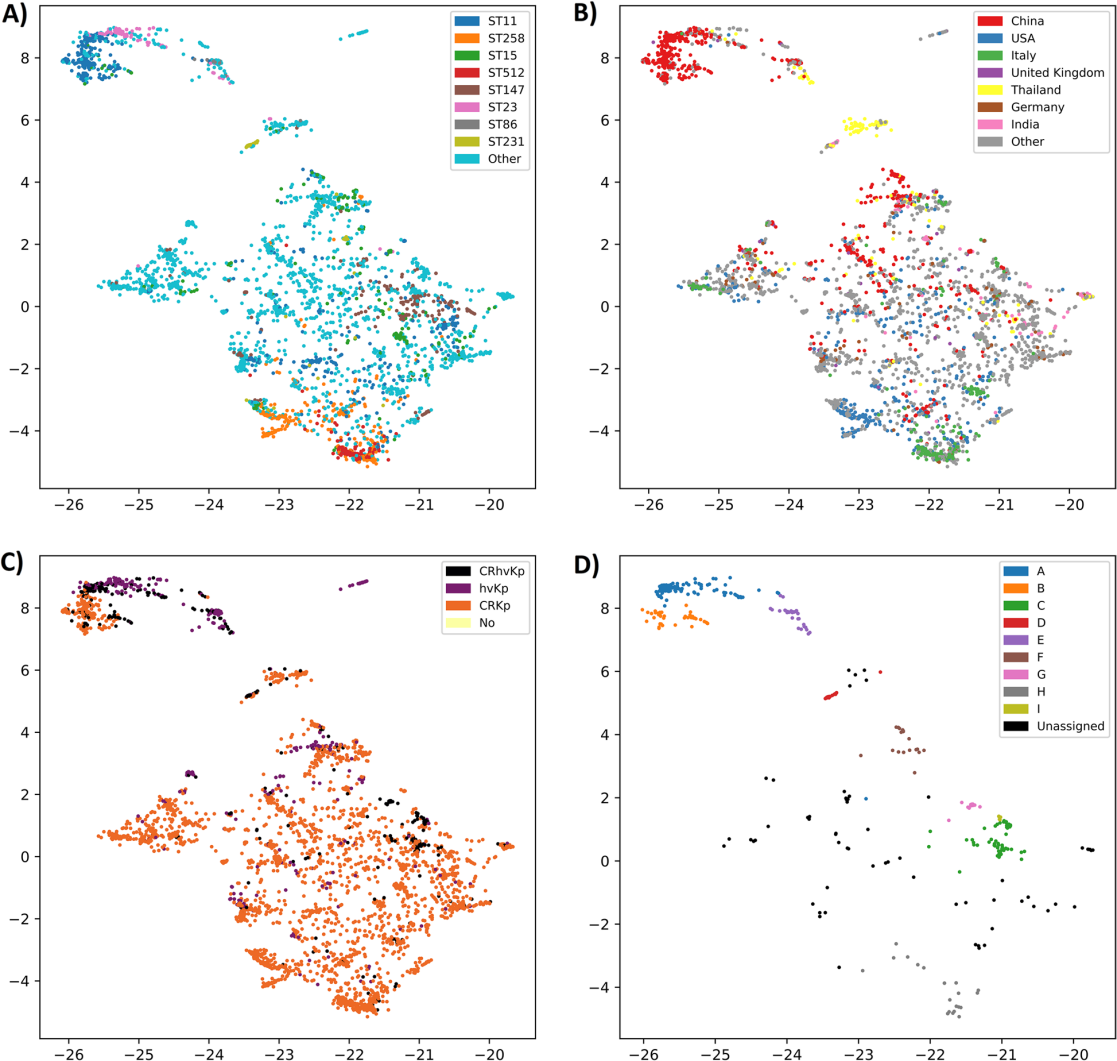
Clustering of CRKp, hvKp, and CRhvKp Kp isolates based on replicon sequences coloured by **A** major sequence types (STs), **B** country, and **C** genotype. In **D**, non-CRhvKp isolates are hidden and only CRhvKp isolates are visible and coloured by plasmid cluster. Axes are dimensionless. Underlying data is presented in Additional file 2: Data S3

To examine the geographical and temporal trends of carbapenem-resistant hypervirulent genotypes, we assessed the replicon-based clustering of CRKp, hvKP and CRhvKp isolates across 695 plasmid replicon nucleotide sequences that were present in multiple CRKp, hvKp and CRhvKp isolates (Additional file 1: Fig. S1). The UMAP-based analysis of population structure revealed that clustering was not driven solely by geography or ST (Fig. 2). This contrasts with chromosomal-based cluster analysis, which revealed clustering of isolates by ST (data not shown). We applied the HDBSCAN clustering algorithm on the two-dimensional UMAP projection of CRhvKp isolates (*n*=1028) and identified 9 groups (Clusters A – I; Table 1, Fig. 2), with 79 (8%) isolates not assigned to any group (see Additional file 2: Data S3 for all assignments). Of these 9 clusters, only two (B and E) did not have strong statistical support of their robustness.

**Table 1:** List of CRhvKp clusters. In some assemblies carbapenemase genes (CR), *iuc* and *iro* (hypervirulence; HV) loci were located on the same assembly contig or chromosome. The underlying data is available in Additional file 2: Data S2, S3 and S4

Cluster	Is robust*	***N***	Main STs (N)	Total STs	Species	Collection dates	Main replicons**	Main countries	Main siderophores	Main carbapenemases	Contig with CR and replicon	Contig with HV and replicon	Contig with CR and HV
**A**	Yes (0%)	560	ST11 (499), ST23 (35), ST268 (13)	11	*K. pneumoniae*, *K. quasipneumoniae*	2012–2021	ColRNAI (943), repB (555), IncFII(pHN7A8) (501), IncHI1B(pNDM-MAR) (489), IncR (486)	China (517), Russia (12), Missing (8), Singapore (6)	iuc 1 (554), iro 1 (90), iro 1 (incomplete) (truncated) (22), iro 1 (incomplete) (55)	OXA-48 (16), KPC-2 (516), IMP-4 (8)	74	341	2
**B**	No (99%)	83	ST11 (74)	5	*K. pneumoniae*	2012–2021	ColRNAI (139), IncFII(pHN7A8) (74), IncR (60)	China (81)	iuc 1 (78)	KPC-2 (79)	20	11	0
**C**	Yes (0%)	82	ST147 (32), ST395 (24), ST383 (10)	15	*K. aerogenes*, *K. pneumoniae*	2012–2020	Col(pHAD28) (91), IncHI1B(pNDM-MAR) (80), IncFIB(pNDM-Mar)(79)	Russia (32), China (16), Egypt (9), Germany (8)	iuc 1 (76)	OXA-48 (51), NDM-1 (23)	16	11	3
**D**	Yes (0%)	61	ST231 (60)	2	*K. pneumoniae*	2013–2019	Col440I (73), IncFIB(pQil) (61), IncFII(K) (61), IncFIA (60), IncFII(pAMA1167-NDM-5) (60), ColKP3 (59), Col(pHAD28) (55)	Thailand (24), India (17), Pakistan (8)	iuc 5 (60)	OXA-232 (59)	60	8	0
**E**	No (88%)	50	ST86 (16), ST65 (14)	10	*K. pneumoniae*	2013–2019	repB (50), IncHI1B(pNDM-MAR) (49)	China (16), Singapore (14), Thailand (13)	iuc 1 (44), iro 1 (40)	KPC-2 (32), OXA-232 (13)	19	32	0
**F**	Yes (0%)	34	ST290 (7), ST15 (6)	12	*K. pneumoniae*	2012–2020	IncFII(K) (35), IncFIB(K) (32)	China (23), Russia (7)	iuc 3 (truncated) (16), iuc 1 (14)	NDM-5 (8), NDM-1 (14), KPC-2 (8)	8	19	2
**G**	Yes (0%)	32	ST15 (26)	6	*K. pneumoniae*	2015–2020	repB (31), ColRNAI (31), IncHI1B(pNDM-MAR) (30), IncFIB(pKPHS1) (28), IncFII(K) (28), ColKP3 (28), Col(pHAD28) (28), Col440I (25), IncFIB(pQil) (21)	China (25)	iuc 1 (32)	OXA-232 (27)	32	7	0
**H**	Yes (0%)	31	ST512 (9)	6	*K. aerogenes*, *K. pneumoniae*	2008–2021	IncFII(K) (23), ColRNAI (22), IncFIB(pQil) (20)	Italy (11), USA (6)	iuc 1 (16), iro unknown (13)	KPC-3 (15)	18	2	0
**I**	Yes (0%)	16	ST395 (16)	1	*K. pneumoniae*	2015–2017	IncHI1B(pNDM-MAR) (16), IncFIB(K) (16), Col440II (16), Col(pHAD28) (16), IncFII(K) (16), IncFIB(pNDM-Mar) (15)	Italy (16)	iuc 1 (15)	KPC-3 (14)	2	5	0
**Unassigned**	Yes (0%)	79	ST101 (8), ST2096 (6), ST218 (6)	25	*K. aerogenes*, *K. variicola* subsp*. variicola*, *K. pneumoniae*	2011–2021	Col(pHAD28) (45), IncFII(K) (40), ColRNAI (38), IncHI1B(pNDM-MAR) (30), IncFIB(pNDM-Mar) (28)	China (16), USA (11), Germany (8), Russia (7)	iuc 1 (38), iuc 5 (7), iro unknown (29), iro 1 (11)	KPC-3 (9), KPC-2(19), OXA-48 (13), OXA-232 (8), OXA-244 (6)	32	17	1

*HV* hypervirulent Kp, *CRKp* carbapenemase-resistant Kp

* Cluster is considered robust if <1% of -log_10_ (chi-squared *p*-value) are <20, see the “Methods” section for detail

** Different clusters with the same main replicons may have different variants

### Replicon clusters

**Fig. 3:**
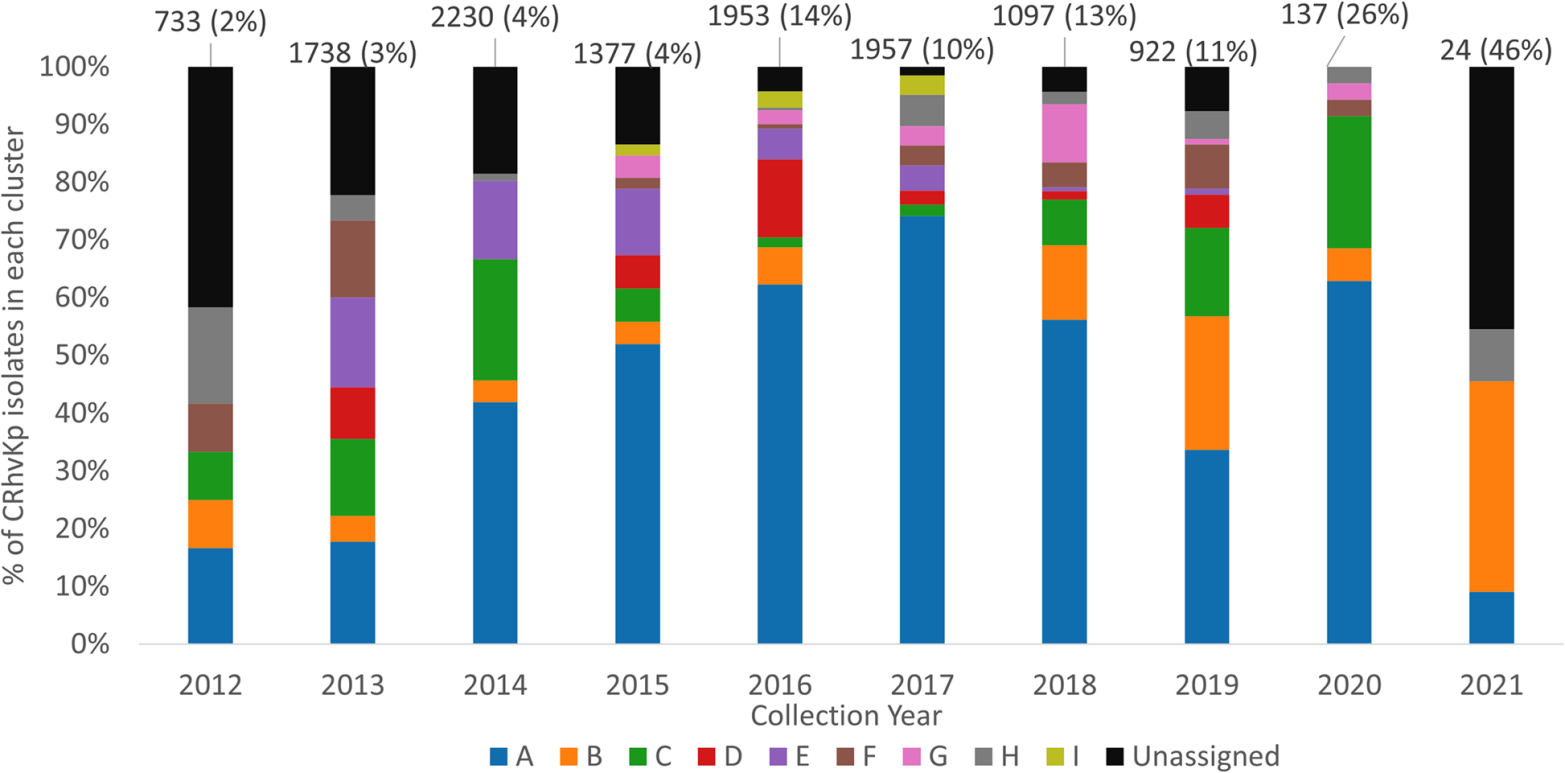
Relative abundance and geographic diversity of carbapenem-resistant hypervirulent Kp (CRhvKp) isolates. Both the total number of all genotyped isolates in each year and percentage that are CRhvKp are presented at the top of the figure

Cluster A (*n*=560, Table 1) consisted mostly of isolates from China and accounted for the majority of CRhvKp isolates from that country (*n*=517/696). This cluster also included isolates with travel links to China [35]. The first isolate from Cluster A was collected in 2012 with the frequency increasing over time (Fig. 3). ST11 was the dominant sequence type in this cluster (*n*=499/560), which also contained the majority of ST11 CRhvKp isolates (*n*=499/582). Hypervirulence was driven by *iuc1* in nearly all isolates (*n*=554/560) and 168 isolates additionally had *iro1* (77 truncated or incomplete), following the nomenclature established previously [36]. The carbapenemase encoding genes were dominated by *bla*_*KPC-2*_ (*n*=516/560), with *bla*_*OXA-48*_ the next most common (*n*=16) (Table 1, Additional file 2: Data S3).

The dominant replicons in Cluster A were ColRNAI (*n*=943), which occurred multiple times in most isolates, followed by repB (*n*=555), IncFII (pHN7A8) (*n*=501), IncHI1B(pNDM-MAR) (*n*=489) and IncR (*n*=486) (Additional file 2: Data S2). By examining assembly contigs for any co-presence of carbapenemase, virulence genes and replicons, we were able to link replicon and carbapenemase genes in 74 isolates and replicon and siderophore genes in 341 isolates (Table 1). No carbapenemase or hypervirulence-linked siderophore genes were found on chromosomes. In cases where siderophores were located on the same contig as replicons, the vast majority (n=290/343) had a IncHI1B(pNDM-MAR) replicon (Additional file 2: Data S4). Of these, 125 contigs had both repB and IncHI1B(pNDM-MAR) replicons. In addition, 42 contigs had simultaneously repB replicon and siderophores. The majority (*n*=52/76) of contigs which carried replicon and carbapenemase genes had an IncFII (pHN7A8) replicon, and 49 of these also had an IncR replicon. Two contigs had both siderophores and carbapenemase genes. The first one had repB, IncFIB (pKPHS1) and IncHI1B(pNDM-MAR) replicons. The second had repB and IncHI1B(pNDM-MAR). Both contigs carried *bla*_*KPC-2*_ and *iuc1* loci.

Cluster B (*n*=83) consisted mainly of isolates from China (*n*=81/83) and the ST11 sequence type (*n*=74/83). This cluster did not have strong statistical support for its robustness, and based on visual examination of results (Fig. 2) it is related to, but distinct from, Cluster A isolates. In particular, the isolates in Cluster B lack repB and IncHI1B(pNDM-MAR) replicons, characteristic of Cluster A.

Cluster C (*n*=82) is geographically diverse, with 32 isolates sourced from Russia, 16 from China, 9 from Egypt, and 8 from Germany. There were 15 STs of which ST147 (*n*=32; 39%) and ST395 (*n*=24; 29%) were the most frequent. While ST147 had broad geographic distribution, the majority of ST395 isolates in this cluster were collected in Russia (*n*=20/24; 83%). The most frequent carbapenemases were *bla*_*OXA-48*_ (*n*=51) and *bla*_*NDM-1*_ (*n*=23), again without any strong geographic links. Five isolates had both genes. More broadly, 78 isolates had either a *bla*_*OXA*_ or *bla*_*NDM*_ gene, with *bla*_*KPC-2*_ (*n*=2) and *bla*_*VIM-1*_ (*n*=1) accounting for the rest (Additional file 2: Data S2). The dominant hypervirulence siderophore was *iuc1* (*n*=78/82), while an unassigned salmochelin lineage was present in two isolates. Despite geographic and ST diversity, three replicons accounted for the majority of isolates: Col(pHAD28) (*n*=91), IncHI1B(pNDM-MAR) (*n*=80), and IncFIB(pNDM-Mar) (*n*=79) (Table 1). Out of 14 isolates in which a replicon and *iuc1* locus were on the same contig, ten had both IncFIB(pNDM-Mar) and IncHI1B(pNDM-MAR) replicons, and a further two had IncHI1B(pNDM-MAR). Replicons linked to carbapenemase encoding genes (*n*=16) were diverse, and IncL linked to *blaOXA-48* (*n*=4) was the most frequent association.

Cluster D (*n*=61) consisted of isolates mainly from South(east) Asia (Thailand, *n*=27; India, *n*=17; Pakistan, *n*=8). The first isolate was collected in Malaysia in 2013, while the most recent six isolates were collected in India in 2019. Nearly all isolates belong to ST231 (*n*=60/61), which shares only two MLST alleles with canonical hypervirulent ST23. The dominant carbapenemase was *bla*_*OXA-232*_ (*n*=59/61), with *bla*_*OXA-181*_ and *bla*_*OXA-48*_ in the other two isolates. Unusually, hypervirulence was driven by *iuc5* (*n*=60/61) and accounts for nearly all *iuc5* carrying CRhvKp (*n*=60/75). Salmochelin (*iro1*) was only present in a single isolate. This cluster had near universal carriage of seven replicons: Col440I, IncFIB(pQil), IncFII(K), IncFIA, IncFII(pAMA1167-NDM-5), ColKP3, and Col(pHAD28) (Table 1). IncFII(pAMA1167-NDM-5) was unique to this cluster and IncFIA only occurred in nine further CRhvKp isolates. We were able to link replicons to a carbapenemase gene in 60 isolates, as well as to a siderophore in 8 isolates. Neither siderophore nor carbapenemase genes occurred on chromosomes. In all cases, *bla*_*OXA-232*_ was linked to the ColKP3 replicon, while *iuc5* was co-located on a contig with IncFIA and IncFII(pAMA1167-NDM-5).

Cluster E (*n*=50) contained isolates mostly from Asia (China, *n*=16; Singapore, *n*=14; Thailand, *n*=13), but unlike Clusters A and B, this one also had five samples from three European countries (Latvia, *n*=2; Greece, *n*=2; France, *n*=1). The first isolates were collected in 2013 in Singapore (*n*=7) and the most recent was from China in 2020. Sixteen isolates were of the canonical hypervirulent strain ST86 and collected in six countries. This cluster also had ST65 (*n*=14) and ST23 (*n*=4) isolates. The former shares only two alleles with ST86. The dominant siderophores in this cluster were *iuc1* (*n*=44) and *iro1* (*n*=40) with 40 isolates carrying both. While *bla*_*KPC-2*_ was the most frequent carbapenemase gene (*n*=32), some isolates (*n*=13) carried *bla*_*OXA-232*_ just like the isolates from Cluster B. All these *bla*_*OXA-232*_ carrying isolates were collected as part of the same Thai study in 2016; however, they belonged to 8 different STs [37]. Unlike Clusters A and B, this one had only two near universal replicons: repB (*n*=50) and IncHI1B(pNDM-MAR) (*n*=49). We were able to link replicon to carbapenemase genes in 19 isolates and to a siderophore in 32 isolates. Nearly all linked *iuc* loci were located on a contig with either IncHI1B(pNDM-MAR) (*n*=4), repB (*n*=1) or both (*n*=22) (Additional file 2: Data S4). The *bla*_*OXA-232*_ gene was co-located with a ColKp3 replicon in all *bla*_*OXA-232*_ carrying isolates, like Cluster B. The replicons linked to *bla*_*KPC-2*_ were much more diverse. Out of 6 contigs three had a IncFII(K) replicon, two IncFII(pHN7A8), and one IncX6. None of the examined contigs carried both carbapenemase and hypervirulence genes, nor were any of these genes on chromosomes.

Cluster G consists of 32 isolates collected every year between 2015 and 2020 with the majority isolated in China (*n*=26) (Table 1). While this cluster includes two ST23 isolates and one ST11, it is dominated by ST15 (*n*=26/32). The main siderophore locus was *iuc1* (*n*=32/32) with two isolates concurrently carrying *iro1*. The dominant carbapenemase was *bla*_*OXA-232*_ (*n*=27/32) with *bla*_*NDM-1*_ (*n*=3/32) next most common. The most interesting aspect of this cluster was the diversity of frequent replicons: repB (*n*=31), ColRNAI (*n*=31), IncHI1B (pNDM-MAR)(*n*=30), IncFIB (pKPHS1)(*n*=28), IncFII(K) (*n*=28), ColKP3(*n*=28), and Col(pHAD28)(*n*=28). We linked replicon to carbapenemase in 32 isolates and to a siderophore in 7 isolates. The *iuc1* hypervirulent locus was linked to IncHI1B(pNDM-MAR) (*n*=6) and blaOXA-232 was linked to ColKP3 (*n*=28) as in cluster D, but that cluster has *iuc5* linked to both IncFIA and IncFII(pAMA1167-NDM-5). Part of this cluster has been described previously [22], reinforcing the robustness of our approach.

### Salmochelin carrying *K. aerogenes*

Surprisingly, nearly all *K. aerogenes* isolates (*n*=288/299) carried a salmochelin locus, but none had aerobactin. In all 41 complete *K. aerogenes* assemblies this locus was chromosomal (contig > 4,500,000nt). These salmochelin genes had nucleotide identity between 74% and 86% to *Kp sensu stricto* salmochelin genes. The nearest amino acid sequences outside *K. aerogenes* were *iroB*, *iroC*, *iroB* and *iroN* in *Enterobacter oligothropicus* with 93%, 90% and 81% identity, respectively. A small portion of *K. aerogenes* isolates had both salmochelin and carbapenemase genes (*n*=40), among which carbapenemase genes *bla*_*KPC-*2_ (*n*=13) and *bla*_*OXA-48*_ (*n*=6) were the most common. Most of the *K. aerogenes* without carbapenemase genes (*n*=231/248) did not have any extended-spectrum beta-lactamase (ESBL) encoding loci, despite most of them (*n*=171/231) being collected after 2010 — a period in which ESBL encoding genes were common in Kp. PlasmidFinder identified replicons in 174 salmochelin carrying *K. aerogenes*. Their replicons formed a cluster of samples consisting mainly of USA isolates (*n*=73), with a few from Germany (*n*=9), Lebanon (*n*=6) and other countries. Apart from one small study [38], we believe this is the first major report of the widespread presence of salmochelin in *K. aerogenes*.

### Comparison to replicons of historic isolates

Plasmid sequences of 35 Kp isolates from the historic Murray collection were identified and compared to the 3034 unique replicons in our whole collection (*n*=12,468). Remarkably, there was a substantial overlap between these unique replicon sequences. For example, the same *repB* sequences occurred in 1124 of all isolates and in 19 Murray collection isolates. Three further replicon sequences [IncFIB(K), IncFII(pKP91) and IncHI1B(pNDM-MAR)] that occurred once each in the Murray collection occurred in more than 240 general isolates (Additional file 2: Data S5). The co-existence of multiple variants of identical replicons up to 90 years ago requires further investigation into the evolution of plasmid replicon sequences, including their mutation rates and any selective pressure.

### IncHI1B(pNDM-MAR) replicon

The Cluster A version of the *repB* gene differs from PlasmidFinder’s IncHI1B(pNDM-MAR) by 4nt and from the closest sequence in our collection by 3nt (out of 570nt). The latter sequence occurs frequently (*n*=395) in our whole dataset (Additional file 2: Data S2). More importantly, the first 96nt of this replicon’s sequence (those preceding a *repB* sequence) are almost unique to Cluster A. These leading 96nt have no similarity using BLAST (word size 7) [39] to any other IncHI1B(pNDM-MAR) variant in our dataset. While this version of replicon does occur outside Cluster A, it is rare among CRhvKp (*n*=3/1028) and CRKp (*n*=4/5763) isolates, but more common among hvKp (*n*=184/988). The Cluster A variant has a strong geographic and ST bias. It is found mostly in China (*n*=561/676), followed by Russia (*n*=22/676) and 18 other countries. KL64 (*n*=343/676) and KL1 (*n*=190/676) are the dominant serotypes, but these are likely the consequence of ST specificity being common in ST23 and ST11 types. Of all ST23 and ST11 isolates with any IncHI1B(pNDM-MAR) replicon, nearly all carried the Cluster A version of the replicon (ST23 197/216; ST11 461/525). In contrast, all isolates of the canonical hypervirulent ST86 (*n*=84/84) carried the PlasmidFinder’s reference replicon variant.

The IncHI1B(pNDM-MAR) replicon was nearly monophyletic in Cluster A, with 473 samples with identical replicon sequences and a further 7 replicon sequences among 18 samples. However, across the entire dataset, the IncHI1B(pNDM-MAR) replicon consisted of five main nucleotide sequences with only six mutation differences (Additional file 2: Data S2). Based on the context of Cluster A’s *bla*_*KPC-2*_ and IncHI1B(pNDM-MAR) replicon nucleotide sequence, there is little evidence that this cluster has generated an epidemic in countries outside of China that are well represented in our dataset. However, within China, Cluster A has been identified in at least 13 provinces since its first isolates were identified in year 2013. The monitoring of the core replicon signatures of such clusters can therefore assist with identifying the spread of CRhvKp forms.

## Discussion

In this study, we examined plasmid replicons in a large global dataset of 12,468 Kp isolates with the aim of understanding the global distribution of CRhvKp isolates. Our analysis revealed there is structure among the 1028 CRhvKp isolates, with most belonging to multi-country clusters, and at least nine clusters present globally. One such cluster (denoted as Cluster A) involved the spread of ST11 CRhvKp isolates and was detected by a surveillance system in China [17], and appears to have been contained in that country. The data also revealed the potential simultaneous spread of a smaller cluster in China involving ST15 strains with plasmid replicons identical to those found in smaller studies from Hangzou and Shanghai regions of China [22, 40]. Other clusters reveal outbreaks in multiple countries, where the exact dynamics of spread are more difficult to determine due to the limited geographic coverage.

We have focused on the plasmid replicons instead of sequence types (STs), because both hypervirulence-associated and carbapenemase-encoding genes are frequently found on plasmids and transfer horizontally. Hypervirulence-associated genes exist almost exclusively on plasmids; while carbapenemase-encoding genes are generally located on small insertion sequences or transposons embedded in plasmids [7, 15]. While STs are important in the context of outbreaks, we think that plasmid replicons provide greater insights into the spread of hypervirulence and carbapenemases, because plasmids are vectors that transfer AMR and virulence genes between bacterial strains. Due to the relative stability of replicon sequences, they can be used as signatures or barcodes of CRhvKp forms for clinical management, surveillance, and infection control activities.

Recent work on CRhvKp epidemiology using genomic data has focused on single hospitals or geographical regions [15, 41]. However, with increased human mobility and travel worldwide, large-scale analyses of all available sequences can provide insights from a global to local resolution. These activities can assist infection control decision-making through identifying transmission hotspots, blind spots of sampling, and informing resource allocation. Despite the global nature of our analysis, there are some geographical gaps due the convenience nature of the sampling, especially with lower numbers of samples contributed from sub-Saharan Africa. While it is difficult to estimate the prevalence of plasmids and replicons, our analysis reveals the presence of their types and diversity, and determines the spread of clusters. The number of actual circulating clusters is likely to be an underestimate and the dynamics of their spread incomplete. Large-scale routine and timely sequencing globally can provide a more complete picture. This process should involve reviewing and updating the core clusters and their signatures, potentially using our statistical approach, which includes consideration of cluster robustness. For identification of links between AMR or hypervirulence loci that are transferred both horizontally and clonally, our methodology could be complementary to the more general and widespread application of phylogenetic trees, which may not be appropriate for *Klebsiella* and other pathogens with horizontal gene transfer [42, 43].

Based on our analysis, heavily represented geographies with clusters of CRhvKp isolates (Thailand, Germany, Italy, USA, Russia) have not demonstrated rapid growth of a single cluster within a country. Instead, there were several small geographically diverse clusters, including one (Cluster H) which has a similar number of cases from Russia, Germany, and Egypt, while another (Cluster I) has isolates mainly from Italy, Russia, and China. Travel linked to tourism to an under-represented country is a plausible explanation. Relatedly, isolates collected across three Russian cities were present in six of nine CRhvKp clusters. This pattern may be linked to travel or importation events.

Another interesting finding was the presence of *K. aerogenes* isolates with salmochelin homologues. This *Klebsiella* species is not common in a clinical setting and generally has a low level of AMR [38], but it may represent a potential reservoir of hypervirulent bacteria, especially in the USA and Germany where it appears most common. Clinical isolates dominate most collections, but the collection and sequencing of samples from environmental or domestic animal sources may provide insights into AMR transmission. Relatedly, the comparison of the replicons from modern and Murray collection isolates showed perfectly matching replicon sequences in several cases, often from isolates spanning 70 to 90 years. While there may be sample fidelity issues arising from long-term storage, this may also be a result of a low mutation rate in replicon sequences. This possibility in turn raises important questions about diversity of plasmids outside clinical environments. Insights into the diversity of plasmids, as well as the emergence and spread of AMR, will be provided through the future widespread application of cost-effective WGS or alternative amplicon sequencing-based approaches across countries and sampling sources, combined with the development of informatic data platforms and large-scale analysis tools.

## Conclusion

Our large-scale analysis of Kp isolates revealed nine global clusters of CRhvKp, including two expanding within China. We found multiple smaller multi-regional clusters that did not have a clear trend in available data. The benefits of the surveillance and monitoring of infections using genomic data have been exemplified by global efforts during the recent COVID-19 pandemic. The generation of sequence data in real time can provide an early warning to infection control decision makers and provide clinical guidance to reduce burden. Relatedly, the presence of multi-country clusters reinforces that the sharing and joint analysis of international data provides important insights into the epidemiology of CRhvKp and other AMR infections, including the identification of countries experiencing outbreaks but potentially without extensive monitoring programmes of their own. The goal would be to identify reservoirs of infections, assist the surveillance activities of infection control agencies, and ultimately reduce the burden of Kp AMR.

## Supplementary Information

**Additional file 1:** **Figure S1.** Analysis work flow, jpg image.

**Additional file 2:** **Data S1.** Samples AMR profile. **Data S2.** Plasmid sequences and their distribution. **Data S3.** Assignments of samples to clusters. **Data S4.** Contig IDs and Replicons. **Data S5.** Murray Collection Replicons

## Data Availability

The datasets supporting the conclusions of this article are available in the NCBI repository. No new isolates were sequenced during this study. Assemblies used are listed in Additional file 2: Data S1. The data was visualised and quantified using a Python Notebook, which is available at https://github.com/AntonS-bio/KpReplicons/ [44].

## Abbreviations

AMR: Antimicrobial resistance
Kp: *Klebsiella pneumoniae*
cKp: Classic *K. pneumoniae*
hvKp: Hypervirulent Kp, defined as carrying either salmochelin or aerobactin locus, but not carbapenemase encoding genes
CRKp: Carbapenemase-resistant Kp, defined as carbapenemase gene carrying Kp, but not salmochelin or aerobactin locus
CRhvKp: Carbapenemase-resistant hypervirulent Kp
MLST: Multilocus sequence type
ST: Sequence type.
