# Supplementary figures and images for: Large-scale genomic analysis of global Klebsiella pneumoniae plasmids reveals multiple simultaneous clusters of carbapenem-resistant hypervirulent strains
Source: Genome Med. 2023 Jan 19;15:3. doi: 10.1186/s13073-023-01153-y (PMC9850321; doi:10.1186/s13073-023-01153-y)

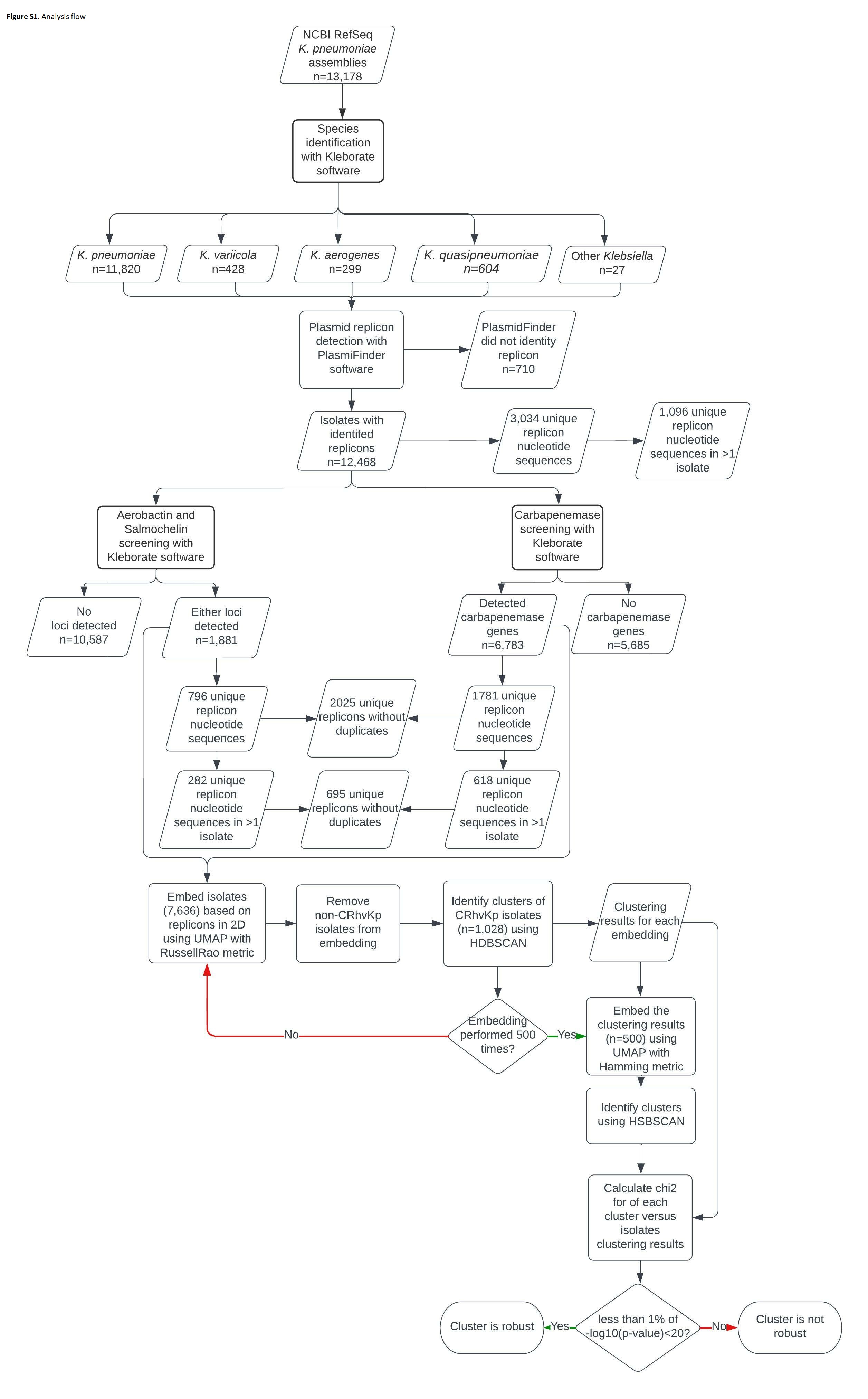

Supplement: Supplementary file 1 — Additional file 1: Figure S1. Analysis work flow, jpg image. [file 13073_2023_1153_MOESM1_ESM.jpeg]
